# Validation of PH and Varices Risk Scores for Prediction of High-Risk Esophageal Varix and Bleeding in Patients with B-Viral Cirrhosis

**DOI:** 10.3390/diagnostics12020441

**Published:** 2022-02-09

**Authors:** Seunghwan Shin, Seung Up Kim, Jun Yong Park, Do Young Kim, Sang Hoon Ahn, Beom Kyung Kim

**Affiliations:** 1 Department of Internal Medicine, Yonsei University College of Medicine, Seoul 03722, Korea; sshhissh@gmail.com (S.S.); ksukorea@yuhs.ac (S.U.K.); drpjy@yuhs.ac (J.Y.P.); dyk1025@yuhs.ac (D.Y.K.); ahnsh@yuhs.ac (S.H.A.); 2 Institute of Gastroenterology, Yonsei University College of Medicine, Seoul 03722, Korea; 3 Yonsei Liver Cancer, Severance Hospital, Seoul 03722, Korea

**Keywords:** liver stiffness, prediction, model, esophageal varix, bleeding, PH risk score, varices risk score, LSPS, comparison, validation

## Abstract

Esophageal varices (EVs) can be accurately predicted using PH and varices risk scores. We aimed to validate their prognostic performances. Methods: We enrolled patients with B-viral cirrhosis as the training cohort (*n* = 503). Areas under receiver operating characteristic curves (AUROCs) for HEV were calculated for PH (=−5.953 + 0.188 × liver stiffness (LS) + 1.583 × sex (1:male/0:female) + 26.705 × spleen diameter/platelet count ratio) and varices (=−4.364 + 0.538 × spleen diameter −0.049 × platelet count −0.044 × LS + 0.001 × LS × platelet count) risk scores, and compared to LSPS (=LS × spleen diameter/platelet count). An independent cohort was recruited for further validation (*n* = 222). In the training cohort, the varices risk score showed the highest AUROC (0.926), followed by the PH risk score (0.924) and LSPS (0.924), but without any statistically significant differences. For varices risk scores ≤−1.70 and ≥1.48, a 95.0% negative predictive value (NPV) and 91.2% positive predictive value (PPV) were observed, respectively. At PH risk scores ≤2.25 and ≥7.71, 95.0% NPV and 90.0% PPV were observed, respectively. At LSPS ≤1.73 and ≥13.9, 95.3% NPV and 95.0% PPV were observed, respectively. The EV bleeding (EVB) risk during follow-up increased stepwise and significantly when stratified by PH, varices risk scores, and LSPS (all *p* < 0.001). In the validation cohort, NPVs were generally similar when stratified by PH (88.2%), varices risk scores (93.2%), and LSPS (88.9%); however, corresponding PPVs were suboptimal. PH and variceal risk scores are reliable for predicting HEV and future EVB. Patients with PH and varices risk scores ≤2.25 and ≤−1.70, respectively, may avoid endoscopy safely. For convenience, LSPS might be a good alternative, with comparable prognostic performance to these two models.

## 1. Introduction

Portal hypertension (PH) is a progressive complication of liver cirrhosis, leading to portosystemic collaterals, such as esophageal varices (EVs) [1,2]. Moreover, EV bleeding (EVB) is one of the most life-threatening complications, and it can accelerate the progression of hepatic decompensation to a stage wherein patients have an extremely high risk of death [3,4]. Therefore, the current guidelines recommend screening all patients with cirrhosis by endoscopy to identify those with high-risk EVs (HEVs), so that prophylactic treatment may be considered [5,6,7]. However, because the prevalence of EVB at any given point in time is approximately 15–25%, most patients undergoing screening endoscopy either do not have varices or have varices that do not require prophylactic therapy [8]. Thus, periodic endoscopic screening in all cirrhotic patients, especially those belonging to the so-called “low-risk group,” might unnecessarily increase the financial burden and medical workload of endoscopy units. Furthermore, compliance with screening endoscopy may be limited because even asymptomatic patients are required to repeatedly undergo an unpleasant endoscopic procedure and an interruption in work productivity, with a small, but significant, risk of complications.

To date, the availability of noninvasive tools has allowed the early identification of asymptomatic patients with liver cirrhosis at the compensated stage, wherein endoscopic screening for gastroesophageal varices is also required. Accordingly, liver stiffness (LS) assessment using transient elastography (TE), as an objective and reliable noninvasive tool to avoid universal screening endoscopy among all cirrhosis patients, has been evaluated for its usefulness to predict the presence of EVs [9,10,11,12,13,14,15,16]. In particular, according to the Baveno IV criteria and its extended criteria, a combination of platelet count and LS value was suggested to be sufficiently adequate to obviate the need for endoscopic screening in patients with compensated advanced chronic liver disease (cALD) [17,18]. In fact, the usefulness of LS–spleen diameter-to-platelet count score (LSPS) has been validated safely, suggesting that universal endoscopic screening can be avoided among patients with hepatitis B virus (HBV)-related cirrhosis or cALD [19,20]. Furthermore, two LS-based models with somewhat complex equations, i.e., PH and varices risk scores, were also introduced as a noninvasive, accurate model to identify the presence of EVs and clinically significant PH of hepatic venous pressure gradient (HVPG) > 10 mmHg [21], confirming that LS alone is a better marker of clinically significant PH, and that its performance can be improved when combined with platelet count and spleen size.

Herein, we aimed to validate the predictive performance of PH and varices risk score for detecting HEV, and to predict future EVB during follow-up, compared to LSPS—a more simplified predictor—among a large cohort of patients with chronic HBV infection.

## 2. Materials and Methods

### 2.1. Study Subjects

Patients presenting with HBV-related liver cirrhosis at the outpatient liver clinic of Severance Hospital, Yonsei University College of Medicine, Seoul, the Republic of Korea, from 2006 to 2014 were considered eligible for this study. After an endoscopic examination, all patients underwent systematic, complete biochemical workups, TE by FibroScan® (Echosens, Paris, France)and abdominal ultrasonography within 1 month. For patients with multiple endoscopy records, the most recent record was used for analysis. The exclusion criteria were as follows: (1) infection with other viral hepatitis; (2) alcohol consumption >30 g/day for more than 5 years; (3) other Child–Pugh class C at enrollment; (4) history of hepatocellular carcinoma; (5) previous history of variceal bleeding, β-blocker therapy, or endoscopic treatments (band ligation and sclerotherapy); (6) previous surgery for PH or trans-jugular intrahepatic portosystemic shunt placement; (7) body mass index >35 kg/m^2^; (8) unreliable LS value. If histologic information was not available, cirrhosis was clinically defined as follows: (1) platelet count <150,000/μL and ultrasonographic findings suggestive of compensated cirrhosis, including a blunted, nodular liver surface, accompanied by splenomegaly (>12 cm), or (2) the presence of esophageal or gastric varices. Furthermore, in order to validate the cut-off determined by applying three noninvasive parameters (PH risk score, varices score, and LSPS), we subsequently enrolled patients who visited the hospital between 2014 and 2017 according to the aforementioned enrollment criteria, as an independent cohort.

### 2.2. Clinical Assessment and Calculation of PH and Varices Risk Scores

EVs were classified into the following three sizes: small (minimally elevated veins above the esophageal mucosal surface), medium (tortuous veins occupying less than one-third of the esophageal lumen), or large (those occupying more than one-third of the esophageal lumen). HEVs were defined as medium/large EVs and small EVs with red signs or decompensated cirrhosis [22]. Within 1 month before or after endoscopy, all the patients underwent laboratory tests, abdominal ultrasonography, and TE. Spleen diameter was defined as the greatest longitudinal dimension at the level of the splenic hilum as measured on the image monitor using electronic calipers. TE was performed using FibroScan^®^ (Echosens, Paris, France). The results were expressed in kilopascals (kPa), and the median value of successful measurements was selected as a representative. Fewer than eight successful acquisitions or a success rate <60% were considered unreliable. Only LS values with an interquartile range (IQR)/median <0.3, at least 10 validated measurements, and a success rate of at least 60% were considered reliable. All endoscopy, TE, and ultrasonography operators were blinded to the patients’ clinical and laboratory data.

Equations (1) and (2) of the two noninvasive LS-based models are as follows:

PH risk score = −5.953 + 0.188 × LS + 1.583 × sex (1: male/0: female) + 26.705 × spleen diameter/platelet count ratio(1)

Varices risk score = −4.364 + 0.538 × spleen diameter − 0.049 × platelet count − 0.044 × LS + 0.001 × (LS × platelet count) (2)

For comparison of the predictive performance of detecting HEVs among the LS-based models, the following was also calculated: LSPS = LS × spleen diameter/platelet count.

### 2.3. Patient Follow-Up

During follow-up, the patients underwent periodic surveillance with endoscopy every 1–3 years, according to the guidelines for screening and follow-up of EVs. Furthermore, they underwent routine blood biochemical tests, assays of serum HBV-DNA levels, and other viral markers every 3–6 months. Abdominal ultrasonography with serum alpha-fetoprotein estimation were also performed every 6 months to detect hepatocellular carcinoma (HCC) for cases in which treatment modalities with a curative intent were considered appropriate [23]. Antiviral therapy using oral nucleos(t)ide analogues was administered to patients with cirrhosis during the follow-up period, if indicated [24,25,26,27]. For patients with HEVs, a non-selective β-blocker, such as propranolol or carvedilol, was also administered as a prophylaxis for EV bleeding (EVB) according to physicians’ discretion, if not contraindicated. Any patient suspected of developing upper gastrointestinal bleeding underwent endoscopic intervention for accurate diagnosis and appropriate treatment. EVB was defined as upper gastrointestinal bleeding confirmed to originate from EVs on endoscopy.

### 2.4. Statistical Analysis

Continuous variables were compared using Student’s *t*-test (or Mann–Whitney test, if appropriate), and categorical variables were compared using chi-square test or Fisher’s exact test. As appropriate, all data are expressed as mean ± standard deviation, median (IQR), and number (%). To assess the prediction performance of HEVs, receiver operating characteristic (ROC) curves were plotted and the area under the ROC curve (AUROC) was computed. Sensitivity, specificity, positive predictive value (PPV), and negative predictive value (NPV) were also calculated using the ROC curves. The cumulative risk of EVB was assessed using the Kaplan–Meier method and compared using the log-rank test. Cox regression analysis was performed to calculate hazard ratios (HRs) with 95% confidence intervals (CIs). Thereafter, to assess the predictive performance of the noninvasive prediction models for cumulative risk of EVB during the follow-up, time-dependent ROC curves were also constructed and AUROCs were computed.

All statistical analyses were performed using R Statistical Software (v.4.0.2.; http://cran.r-project.org/, accessed on 31 January 2022). Two-sided *p*-values <0.05 were considered indicative of a statistically significant difference.

## 3. Results

### 3.1. Baseline Characteristics among the Training Cohort

For the training cohort, 503 patients with HBV-related liver cirrhosis were enrolled. Table 1 presents the baseline characteristics of the study population. The mean age of the patients was 53.0 years, with a male predominance of 69.0%. Most patients had Child–Pugh class A disease (96.2%). A total of 209 patients (41.6%) had EVs, consisting of 72 small, 104 medium, and 32 large varices, and HEV was observed in 146 patients (29.0%). The mean LS value was 22.6 ± 18.8 kPa, and the mean spleen diameter was 11.6 ± 2.6 cm.

### 3.2. Predictive Performances of Noninvasive Models to Detect the HEVs and Determine the Useful Cutoff Value in the Training Cohort

**Figure 1:**
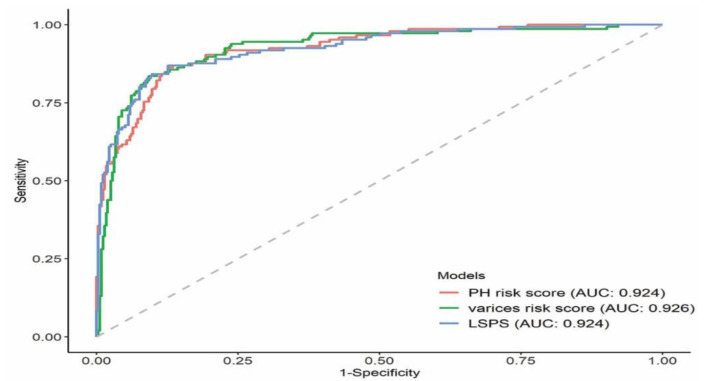
Predictive performance of the three noninvasive models to detect the presence of HEV in the training cohort.

To compare the predictive performance of noninvasive models for the presence of HEVs, we calculated the AUROCs of three noninvasive models—PH risk score, varices risk score, and LSPS (Figure 1). The AUROC of the varices risk score (0.926; 95% confidence interval (CI): 0.900–0.953) was the highest, followed by the LSPS (0.924, 95% CI: 0.897–0.951), and PH risk score (0.924; 95% CI: 0.898–0.949). However, among the three models, there was no statistically significant difference in terms of AUROCs between each pair; *p* = 0.876 between LSPS and PH risk score, *p* = 0.785 between LSPS and varices risk score, and *p* = 0.697 between PH and varices risk score. In the same cohort, we used the following cutoffs for each model to achieve an NPV and PPV of approximately ≥95% and ≥90%, respectively (Table 2): PH risk score, ≤2.25 and ≥7.71; varices risk score, ≤−1.70 and ≥1.48; LSPS, ≤1.73 and ≥13.9.

### 3.3. Predictive Performances of Noninvasive Models to Detect the HEVs in the Validation Cohort

Supplementary Table S1 shows the baseline characteristics of the validation cohort (*n* = 222). When the predictive performance of the three models was calculated in the validation cohort, the AUROC of the varices risk score (0.803, 95% CI: 0.742–0.863) was the highest, followed by the LSPS (0.795, 95% CI: 0.733–0.856), and PH risk score (0.788, 95% CI: 0.724–0.853), without any statistically significant differences (all *p* > 0.05).

In addition, Table 3 shows the NPVs and PPVs in the validation cohort, obtained using the above-suggested cutoffs; these values were calculated to achieve an NPV of ≥95% and PPV of ≥90% in the training cohort. The comparable NPVs of 93.2%, 88.2%, and 88.9% were reproduced using a varices risk score ≤−1.70, PH risk score ≤2.25, and LSPS ≤1.73, respectively. However, in terms of PPVs, the three prediction models consistently showed the following relatively suboptimal predictive performances: 62.2% with PH score ≥ 7.71, 45.8% with varices risk score ≥ 1.48, and 83.3% with LSPS ≥ 13.9.

### 3.4. EVB Risk during Follow-Up in the Training Cohort

**Figure 2:**
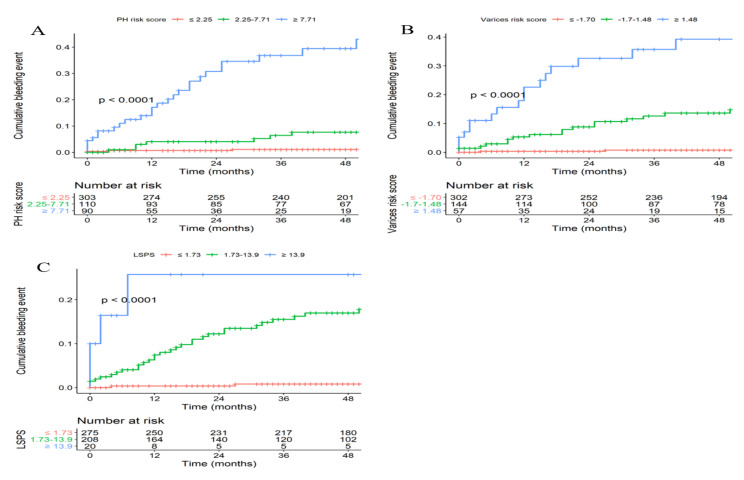
Cumulative risk of EV bleeding based on suggested cutoffs by the PH risk score (**A**), varices risk score (**B**), and LSPS (**C**) in the training cohort.

During the median follow-up period of 51 (IQR, 23.0–74.0) months, 40 (8.0%) patients experienced their first EVB episodes. The cumulative risk of EVB at 1, 2, 3, and 4 years was 4.1%, 6.0%, 7.5%, and 8.1%, respectively. When the risk of EVB was assessed according to the suggested cutoffs of PH, varices risk score, and LSPS, the EVB risk increased significantly in a stepwise manner (all *p* < 0.001; Figure 2A–C). Compared to patients with a PH risk score ≤2.25, those with a PH risk score ≥7.71, and between 2.25 and 7.71, had a significantly higher risk of future EVB, with an HR of 25.63 (95% CI: 10.47–62.77) and 3.93 (95% CI: 1.36–11.32) (both *p* < 0.001), respectively. Similarly, compared to patients with a varices risk score <−1.70, those with a varices risk score ≥1.48, and between −1.70 and 1.48, had a significantly higher risk of future EVB, with an HR of 48.34 (95% CI: 14.12–165.45) and 15.95 (95% CI: 4.74–53.70) (both *p* < 0.001), respectively. Finally, compared to those with an LSPS ≤1.73, those with an LSPS ≥13.9 and 1.73–13.9 showed a significantly greater risk of EVB, with an HR of 40.58 (95% CI: 9.03–182.44) and 17.21 (95% CI: 5.28–56.13) (both *p* < 0.001), respectively. 

The areas under the time-dependent ROC curves of the three models to predict future EVB events at 24 and 48 months showed relatively high levels (almost 0.9; Table 4). In terms of the predictive performance of EVB at 48 months, the PH risk score was a better predictor than the varices risk score, with a marginal difference (*p* = 0.0497). However, there was no statistically significant difference between the three models at 24- and 48-month follow-ups (all *p* > 0.05).

## 4. Discussion

Since EVB is a life-threatening condition, necessitating emergent approaches, endoscopic surveillance should be performed every 2–3 years among patients with compensated cirrhosis, and annually among those with decompensated cirrhosis, both of which allow timely prophylaxis [1]. Although endoscopy is a gold standard approach to screen for EVs, it can potentially cause discomfort and complications, due to its invasiveness. Furthermore, an individual’s EVB risk differs markedly according to their fibrotic burden and hepatic functional reserve; thus, mortality rates vary from 3.4% to ≥20% per year. Therefore, noninvasive screening tools to identify high-risk patients have been suggested.

In the present study, both PH and varices risk scores showed excellent AUROCs for the presence of HEV, nearly equivalent to that of LSPS. Three key components—LS value, spleen diameter, and platelet count—theoretically have a good correlation with PH [19,28]. When cirrhotic transformation increases, liver tissue stiffness, caused by major angio-architectural modifications, and the accumulation of the fibrillar extracellular matrix occurs, leading to portal hypertension [10]. Splenomegaly is most likely due to vascular disturbances, and is almost always an expression of greater portal pressure, while thrombocytopenia might be caused by either portal hypertension or other mechanisms, such as decreased thrombopoietin, shortened platelet lifetime, myelotoxic effects, or the hepatitis virus [14]. In particular, spleen size alone had considerable efficiency in diagnosing HEV, with an AUROC of 0.900 (95% CI 0.870–0.929) with a suggested threshold of 12.0 cm as Youden’s index. We observed that HEVs could be excluded with an NPV of approximately 95.0%, using the following cutoff values: PH risk score ≤2.25 (*n* = 303), varices risk score ≤−1.70 (*n* = 302), and LSPS ≤1.73 (*n* = 275); this finding indicates that endoscopic surveillance could be confidently exempted in approximately 60% of patients, without a significant risk of missing HEVs. For such a low-risk group, only periodic follow-up with noninvasive markers may be sufficient. In the validation cohort, using the suggested cutoffs of ≤2.25 for PH risk score, ≤−1.70 for varices risk score, and ≤1.73 for LSPS, the varices risk score showed the closest NPV to a target of 95% (93.2%), whereas the PH risk score and LSPS achieved NPVs of about 90%. Moreover, with a PH risk score ≥7.71, varices risk score ≥1.48, and LSPS ≥13.9, PPVs of 90.0%, 91.2%, and 95.0%, respectively, were achieved. Notably, in the validation cohort, the PPVs from the three models were consistently suboptimal, at 62.2%, 45.8%, and 83.3%, respectively. Therefore, we cautiously conclude that the major role of these noninvasive prediction models should be to identify a specific subgroup in which endoscopic surveillance can be safely avoided. Regarding future EVB risk, the three models showed similar areas under the time-dependent ROC curves (almost 0.9) and showed good predictive power in the follow-up periods of 24 and 48 months. Similarly, the predictive performance of future EVB events was generally comparable among the three models. However, from a practical point of view, given the intricate equations for both PH and varices risk scores, the simpler LSPS, with comparable performance among all three models, might be a useful alternative in the outpatient clinic or at the patient’s bedside.

Our study had several strengths. Firstly, the relatively large sample size of >500 patients with liver cirrhosis, during the long-term follow-up of a median of 51.0 months, enhanced the statistical reliability of the results, conferring adequate statistical power. Secondly, the distribution of our study population was homogenous, in terms of ethnicity and etiology, representative of patients with B-viral liver cirrhosis in the Republic of Korea. Our study might provide more generally applicable results, at least for HBV-related liver cirrhosis, considering that the LS value is known to have different cutoff values to determine the severity and diagnostic efficacies according to the underlying etiology. Finally, for more independent validation, we additionally established a cohort with an appropriate sample size (*n* = 222) who visited the hospital between 2014 and 2017, according to the same enrollment criteria.

This study also has several limitations. Firstly, since it utilized a retrospective design and patients from a single hospital, selection bias might have occurred. In particular, detailed information regarding the amount of alcohol consumed and other lifestyle factors was not available, due to the retrospective study design. Thus, in order to overcome such shortcomings, a prospective study based upon a multi-center cohort would be required. Secondly, HVPG, as a standard method to assess portal pressure, was not measured in this study [13]. Nevertheless, a reliable correlation between LS and HVPG has been widely reported in many studies [29,30,31]. Thirdly, LS-based, noninvasive models have inherent limitations for patients with severe ascites or obesity. However, among the population in the Republic of Korea, reliable LS values were unavailable for only a minor portion [28]. Fourthly, since we limited the study population to patients with HBV-related cirrhosis, the conclusions from our study might not be generalizable to the full spectrum of patients with liver cirrhosis. Further studies are required to validate our results with other etiologies, especially non-alcoholic steatohepatitis, which has become the predominant cause of liver cirrhosis in developed countries [32]. Fifthly, the use of new HBV-specific biomarkers that can reflect the clinical stage of chronic HBV infection more precisely would have allowed more detailed analyses [33,34,35,36,37,38,39]. Lastly, owing to the relatively small size of EVB (*n* = 40, 8.0%), the predictive performance for a future bleeding event might be compromised. Further studies with longer follow-up durations, enabling the observation of a sufficient number of events, may be required.

## 5. Conclusions

Both PH and varices risk scores are reliable, noninvasive models for detecting HEVs and predicting future EVB among patients with B-viral liver cirrhosis. Accordingly, approximately 60% of patients may safely avoid endoscopy. Furthermore, for convenience, LSPS, a simplified equation with comparable prognostic performance, might be a good alternative in clinical settings.

## Figures and Tables

**Table 1:** The baseline clinical characteristics of the training cohort (*n* = 503).

Variables	Values
Age, years	53.0 ± 8.9
Male	347 (69.0)
Presence of HEV	146 (29.0)
Child–Pugh class A/B	484 (96.2)/19 (3.8)
Ascites	82 (16.3)
AST, U/L	47.1 ± 50.8
ALT, U/L	43.8 ± 57.4
Albumin, g/dL	4.2 ± 2.0
Total bilirubin, mg/dL	1.6 ± 3.7
PT-INR	1.1 ± 0.2
Platelet count, ×10^9^/L	129.6 ± 63.3
LS, kPa	22.6 ± 18.8
Spleen diameter, cm	11.6 ± 2.6
LSPS	3.3 ± 4.2
PH risk score	2.7 ± 5.1
Varices risk score	−2.9 ± 3.7

Values are expressed as mean ± standard deviation or no. (%). Abbreviations: HEV, high-risk esophageal varix; AST, aspartate aminotransferase; ALT, alanine aminotransferase; PT, prothrombin time; INR, international normalized ratio; LS, liver stiffness; LSPS, liver stiffness–spleen diameter-to-platelet ratio score; PH, portal hypertension.

**Table 2:** Diagnostic performances to predict the presence of HEV (*n* = 146) by the suggested cutoff values of each model among the training cohort.

Cutoff Values	NPV (95% CI)	PPV (95% CI)	Sensitivity (95% CI)	Specificity (95% CI)
PH score ≤ 2.25	95.0% (92.0–97.2)	65.5% (58.5–72.1)	89.7% (83.6–94.1)	80.7% (76.2–84.6)
PH score ≥ 7.71	84.3% (80.4–87.6)	90.0% (81.9–95.3)	55.5% (47.0–63.7)	97.5% (95.3–98.8)
Varices score ≤ −1.70	95.0% (91.9–97.2)	65.2% (58.2–71.7)	89.7% (83.6–94.1)	80.4% (75.9–84.4)
Varices score ≥ 1.48	78.9% (74.8–82.6)	91.2% (80.7–97.1)	35.6% (27.9–44.0)	98.6% (96.8–99.5)
LSPS ≤ 1.73	95.3% (92.1–97.5)	58.3% (51.6–64.8)	91.1% (85.3–95.2)	73.4% (68.5–77.9)
LSPS ≥ 13.9	73.7% (69.5–77.6)	95.0% (75.1–99.9)	13.0% (8.0–19.6)	99.7% (98.4–100)

Abbreviations: HEV, high-risk esophageal varix; NPV, negative predictive value; PPV, positive predictive value; CI, confidence interval; PH, portal hypertension; LSPS, liver stiffness–spleen diameter-to-platelet ratio score.

**Table 3:** The predictive performances produced by the cutoffs from each model in the validation cohort.

Scoring Model	NPV ≥ 95% (Cutoff)	PPV ≥ 90% (Cutoff)
PH risk score	88.2% (≤2.25)	62.2% (≥7.71)
Varices risk score	93.2% (≤−1.70)	45.8% (≥1.48
LSPS	88.9% (≤1.73)	83.3% (≥13.9)

Abbreviations: NPV, negative predictive value; PPV, positive predictive value; PH, portal hypertension; LSPS, liver stiffness–spleen diameter-to-platelet ratio score.

**Table 4:** AUC of time-dependent ROC curves of the three models to predict EVB at 24 and 48 months.

Scoring Model	24 Month	48 Month
PH risk score	91.7%	91.0%
Varices risk score	89.2%	88.2%
LSPS	91.0%	90.1%

Abbreviations: AUC, area under curve; ROC, receiver operating characteristic; EVB, esophageal varix bleeding; PH, portal hypertension; LSPS, liver stiffness–spleen diameter-to-platelet ratio score.

## Data Availability

The data presented in this study are available on request from the corresponding author. The data are not publicly available due to patient privacy concerns.

## Supplementary Materials

Supplementary Table S1 can be found at https://www.mdpi.com/article/10.3390/diagnostics12020441/s1.
