# Supplementary material for: Validation of PH and Varices Risk Scores for Prediction of High-Risk Esophageal Varix and Bleeding in Patients with B-Viral Cirrhosis
Source: Diagnostics (Basel). 2022 Feb 9;12(2):441. doi: 10.3390/diagnostics12020441 (PMC8870789; doi:10.3390/diagnostics12020441)
Supplement: Supplementary file 1 [file diagnostics-12-00441-s001.zip › diagnostics-1555521-supplementary.pdf]

Supplementary Table S1. The baseline clinical characteristics of the validation cohort (*N* = 222).

Variables	Values
Age, years	61.1 ± 9.6
Male	149 (67.1)
Presence of HEV	59 (26.6)
Child-Pugh class A/B	205 (92.3) / 17 (7.7)
Ascites	31 (14.0)
AST, U/L	49.8 ± 70.2
ALT, U/L	40.8 ± 64.6
Albumin, g/dL	3.8 ± 0.7
Total bilirubin, mg/dL	1.5 ± 2.4
PT-INR	1.1 ± 0.2
Platelet count, $\times 10^9$/L	114.7 ± 61.3
LS, kPa	19.1 ± 16.7
Spleen diameter, cm	11.9 ± 2.2
LSPS	3.3 ± 4.9
PH risk score	2.7 ± 4.9
Varices risk score	−2.5 ± 3.4

Values are expressed as mean ± standard deviation or no. (%). **Abbreviations:** HEV, high-risk esophageal varix; AST, aspartate aminotransferase; ALT, alanine aminotransferase; PT, Prothrombin time; INR, international normalized ratio; LS, liver stiffness; LSPS, liver stiffness-spleen diameter to platelet ratio score.
